# Complete Genome of Hepatitis E Virus from Laboratory Ferrets

**DOI:** 10.3201/eid2004.131815

**Published:** 2014-04

**Authors:** Tian-Cheng Li, Tingting Yang, Yasushi Ami, Yuriko Suzaki, Masayuki Shirakura, Noriko Kishida, Hideki Asanuma, Naokazu Takeda, Wakita Takaji

**Affiliations:** National Institute of Infectious Diseases, Tokyo, Japan (T.-C. Li, Y. Ami, Y, Suzaki, M. Shirakura, N. Kishida, H. Asanuma, W. Takaji);; Affiliated Hospital of Qingdao University Medical College, Qingdao, China (T. Yang);; Osaka University, Osaka, Japan (N. Takeda)

**Keywords:** hepatitis E virus, HEV, viruses, ferrets, laboratory ferrets, imported infection, complete genome, sequencing, zoonoses

## Abstract

The complete genome of hepatitis E virus (HEV) from laboratory ferrets imported from the United States was identified. This virus shared only 82.4%–82.5% nt sequence identities with strains from the Netherlands, which indicated that the ferret HEV genome is genetically diverse. Some laboratory ferrets were contaminated with HEV.

Hepatitis E virus (HEV) is a single-stranded positive-sense RNA virus that belongs to the family *Hepeviridae*, genus *Hepevirus*, and is the causative agent of hepatitis E (*1**,**2*). Because the transmission of HEV from deer, swine, and wild boars to humans is well known, hepatitis E is recognized as a zoonosis. Zoonotic hepatitis E is associated mainly with genotype 3 (G3) and G4 HEV infection (*3**,**4*). In addition to deer, swine, and wild boars, other animals, including monkeys, rats, ferrets, chickens, and bats, harbor HEV or HEV-like viruses (*5**–**9*). The genus *Hepevirus* might include 3 additional species (avian HEV, bat HEV, and rat/ferret HEV) (*10*). However, whether HEV from these animals is transmitted to humans is not clear.

HEV has been detected in ferrets (*Mustela putorius*) in the Netherlands (*6*). The ferret HEV genome contains 3 open reading frames (ORFs 1–3). ORF1 encodes a nonstructural protein of 1,596; ORF2 encodes a capsid protein of 654 aa, and ORF3 encodes a functionally unknown phosphoprotein of 108 aa. A putative ORF4 observed in the ferret HEV genome was also found in the rat HEV genome. Nucleotide sequence analyses indicated that the ferret HEV genome shares the highest nucleotide sequence identity (72.3%) with rat HEV. The nucleotide sequence identity between the ferret HEV and G1–4 HEV, rabbit HEV, and avian HEV ranges from 54.5% to 60.5% (*6*). However, the antigenicity, pathogenicity, and epidemiology of ferret HEV remain unclear.

Ferret HEV was also recently detected in the United States in serum (*11*), suggesting that ferret HEV infection is not restricted to the Netherlands and might be distributed in ferrets worldwide. Because ferrets are susceptible to several respiratory viruses, including human and avian influenza viruses, and severe acute respiratory syndrome coronavirus (*12**,**13*), ferrets have been used as a small-animal model for these viruses. Ferrets are also kept as pets in many countries. Thus, information about ferret HEV epidemiology, distribution, transmission, and pathogenesis is urgently needed.

In this study, we amplified and analyzed the complete genome of the US strains of ferret HEV to confirm whether US strains are new ferret HEV genotypes. Phylogenetic analysis demonstrated that HEVs detected in laboratory ferrets from the United States are genetically different from those detected in the Netherlands, suggesting that the ferret HEV genome is genetically diverse.

## The Study

Sixty-three fecal samples were collected from laboratory ferrets (*Mustela putorius furo*) at the National Institute of Infectious Diseases, Tokyo, Japan, on May 24, 2013. These ferrets had been imported from a farm in the United States for influenza research 7 days before sample collection. Fecal specimens were diluted with 10 mmol/L phosphate-buffered saline to prepare a 10% suspension, shaken at 4°C for 1 h, and clarified by centrifugation at 10,000 × *g* for 30 min. The supernatant was passed through a 0.45-µm membrane filter (Millipore, Bedford, MA, USA), and stored at −80°C until use.

RNA was extracted by using the MagNA Pure LC Total Nucleic Acid Isolation Kit (Roche Applied Science, Mannheim, Germany) according to the manufacturer’s recommendations. Reverse transcription was performed by using the Superscript II RNase H^−^ reverse transcription procedure (Invitrogen, Carlsbad, CA, USA) and primer TX30SXN as described (*14*). Ferret HEV RNA was detected by using a nested, broad-spectrum reverse transcription PCR (*15*). Forty (63.5%) of 63 fecal specimens were positive for ferret HEV RNA. Sequences were similar to those detected in ferret serum samples in the United States (*11*), which suggested that the laboratory ferrets were infected in the United States and then transported to Japan.

RNA from 2 ferret HEVs was randomly selected, and the full-length genome was amplified by using reverse transcription PCR with primers based on nucleotide sequences derived from strains from the Netherlands and United States (Table 1). Sequence of the 5′-terminal noncoding regions of the genome was determined by using Rapid Amplification of cDNA Ends Kits (Invitrogen) according to the manufacturer’s instructions. All PCR products were purified by using the QIAquick PCR Purification Kit (QIAGEN, Valencia, CA, USA) and cloned into the TA cloning vector pCR2.1 (Invitrogen). Nucleotide sequencing was conducted by using an ABI 3130 Genetic Analyzer Automated Aequencer (Applied Biosystems, Foster City, CA, USA).

**Table 1:** Oligonucleotides used to amplify ferret hepatitis E viruses

Primer (nucleotide positions)*	Sequence, 5′→3′	Product length, bp†
Forward FF1 (1–21)	GGCAGACCCCTAATGGAGACA	
Reverse FR628 (628–648)	GTTGCGTGCGACATAGGCCTT	626
Forward FF541 (541–561)	AGCAATGTATCGCCATGGCAT	
Reverse FR1535 (1535–1554)	ATCTGCATCAGTCGGGCACA	1,014
Forward FF1518 (1518–1538)	AGGATCTGACAGTAGACCTGT	
Reverse FR2555 (2555–2577)	TGCAATGCCAAATTAGCTGTGT	1,060
Forward FF2401 (2401–2421)	GGCGATGAGTTGTACCTGTTA	
Reverse FR3424 (3432–3445)	GAGCAGCCGGTAACATACTCAA	1,045
Forward FF3336 (3336–3355)	GCACAATTTCTATCTCACCA	
Reverse FR4210 (4210–4230)	ACTCCGAATCAGATGATACA	985
Forward FF4181 (4181–4202)	GGCTGGTGCACCTGAATGGCT	
Reverse FR5800 (5800–5821)	TCAGGCAGACGGCGTATCTTAT	1,641
Forward FF4812 (4812–4831)	ATGGAGCATGTGTACAAGAT	
Reverse TX30SXN	GACTAGTTCTAGATCGCGAGCGGCCGCCCTTTTTTTTTTTTTTTTTTTTTTTTTTTTTT	≈2,050
Reverse FR451‡	ACACCGTGTGAATCCCTCCGT	
Abridged amplification	GGCCACGCGTCGACTAGTACGGGIIGGGIIGGGIIG	
Reverse FR279 (279–300)	ATAGATCTAGGATGCGCACCAA	§
Abridged universal amplification	GGCCACGCGTCGACTAGTAC	
Reverse FR191 (191–211)	CGGATGCGACCAAACAACAGA	≈240

*Values in parentheses indicate positions of the primer corresponding to the entire genome of hepatitis E virus (JN998607) isolated from ferret. †Blank cell indicates that 1 primer pair produced 1 product.  ‡Used only for cDNA synthesis. §PCR product was not detected.

Both ferret HEV genomes consisted of 6,820 nt and a poly (A) tail (GenBank accession nos. AB890001 and AB890374), and nucleotide sequence identity was 99.6%. Genomic structure of strains from the United States was similar to that of 2 strains from the Netherlands. The amino acid alignment of ORF2 indicated that ferret HEV ORF2 has an additional 6 amino acids at the N terminus. However, because the seventh codon is AUG, it is unclear which codon was used for the ORF2 translation initiation.

The ORF1 of strains from the United States encodes 1,589 aa, which is 7 aa shorter than ORF1 of both strains from the Netherlands. In addition, the ferret HEV strains from the United States have 2 aa insertions between amino acid residues 596–597 (Thr) and 631–632 (Ile) and 9 aa deletions in amino acid residues 645–653 (Cys-Leu-Arg-Ser-Ser-Pro-Lys-Pro-Pro), which corresponds to those of strains from the Netherlands. Similar to ferret HEV from the Netherlands, an additional putative 183-aa ORF 4 (nt 30–581) was found in strains from the United States. Analysis of 5 other entire ORF2 sequences (GenBank accession nos. AB890375–AB890379) showed that nucleotide identities among them were 98.9%–99.5%, which indicated that genetically similar ferret HEV strains had circulated at the ferret farm in the United States.

Nucleotide and deduced amino acid sequence identities between ferret HEV from the United States and other HEVs are shown in Table 2. The entire genome of strains from the United States shared relatively high nucleotide sequence identities (82.4%–82.5%) with strains from the Netherlands. We generated phylogenetic trees based on ORF2 or the entire genome. These trees showed that although strains from the United States were closely related to strains from the Netherlands, they formed a new and distinct cluster (Figure). We observed similar phylogenetic clustering when we analyzed nucleotide sequences of ORF1 and ORF3 separately. Although we cannot conclude whether ferret HEV from the United States is a new genotype, these results indicated that there is genetic variety in ferret HEV. Researchers should also bear in mind that some laboratory ferrets are contaminated with ferret HEV.

**Table 2:** Nucleotide and deduced amino acid sequence identities between ferret HEV from the United States and other HEVs*

HEV strain (GenBank accession no.)	Full-length genome, %	Ferret HEV (AB890374)					
		Nucleotides, %			Amino acids, %		
		ORF1	ORF2	ORF3	ORF1	ORF2	ORF3
Genotype 1 (NC-001434)	53.6	51.4	58.9	50.3	51.4	56.9	22.2
Genotype 2 (M74506)	53.8	51.7	59.3	49.4	56.0	57.2	25.2
Genotype 3 (AF060668)	55.2	53.6	59.4	47.9	54.5	58.1	22.9
Genotype 4 (AJ272108)	53.9	51.5	59.5	49.4	53.5	57.5	28.8
Wild boar HEV (AB573435)	54.4	51.9	60.5	49.1	57.2	57.3	24.3
Wild boar HEV (AB602441)	54.0	51.9	59.1	50.9	57.4	57.0	31.5
Rabbit HEV (FJ906895)	54.8	57.0	60.5	51.2	55.2	57.7	23.3
Rat HEV (GU345042)	61.2	57.0	71.3	63.3	70.1	79.4	40.4
Rat HEV (JX120573)	62.6	58.1	72.8	64.9	74.1	80.0	43.5
Ferret HEV (998606)	82.4	81.4	84.9	85.9	91.5	94.2	73.1
Ferret HEV (998607)	82.4	81.3	85.1	85.9	91.3	94.8	73.1
Ferret HEV (AB890001)	99.6	99.7	99.5	100.0	99.7	99.8	100.0
Avian HEV (AY535004)	50.8	50.5	54.2	47.0	43.1	47.9	24.2
Bat HEV (JQ001749)	46.8	49.7	54.7	48.8	44.6	54.3	42.9
*HEV, hepatitis E virus; ORF, open reading frame.							

**Figure:**
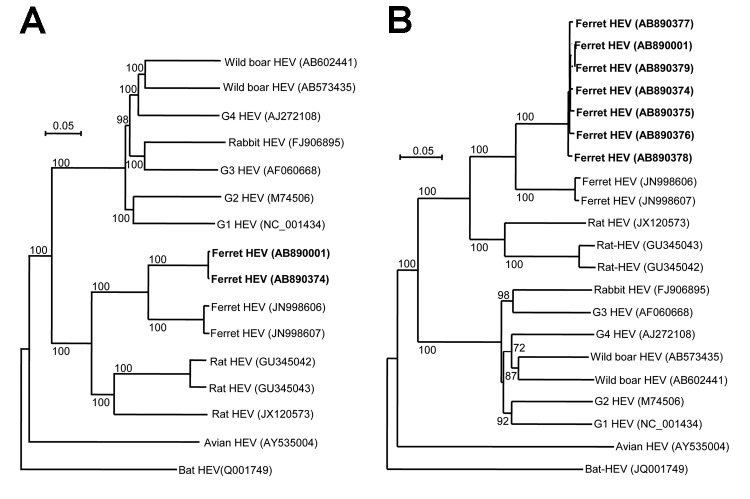
Phylogenetic relationships among genotypes 1–4 and wild boar, rabbit, rat, avian, bat, and ferret isolates of hepatitis E virus. Nucleic acid sequence alignment was performed by using Clustal X 1.81 (www.clustal.org/clustal2/). Genetic distance was calculated by using Kimura’s 2-parameter method. Phylogenetic trees with 1,000 bootstrap replicates were generated by using the neighbor-joining method (Njplot 2.3, http://njplot.sharewarejunction.com/) based on A) the entire genome and B) open reading frame 2. Items in boldface indicate strains isolated in this study. Numbers along branches indicate bootstrap values. Scale bars indicate nucleotide substitutions per site.

## Conclusions

We amplified the entire genome of 2 ferret HEV strains isolated from laboratory ferrets imported from the United States. Nucleotide sequence comparisons showed that 2 ferret HEV strains from the United States had high (99.6%) identity and shared 98.6%–100% identities with partial sequences of ORF1 that were detected in the United States (*11*), which indicated that genetically similar ferret HEV was circulating in laboratory ferrets.

Although nucleotide sequence identities of the entire genome for strains from the United States and the Netherlands was 82.4%–82.5%, ORF2 showed relatively high amino acid identities (94.2%–94.8%), which suggested that isolated from the United States and the Netherlands share similar antigenicity. Ferret HEV–like particles derived from 1 of the isolates from the Netherlands were cross-reactive with serum from HEV-infected laboratory ferrets in the United States (*11*).

In conclusion, we isolated and identified 2 ferret HEV strains from laboratory ferrets imported from the United States. These strains were genetically distinct from ferret HEV isolates from the Netherlands. Some laboratory ferrets were contaminated with ferret HEV. Further studies are needed to confirm the pathogenicity and zoonotic potential of ferret HEV.
